# Association between Macronutrient Intake and Excessive Daytime Sleepiness: An Iso-Caloric Substitution Analysis from the North West Adelaide Health Study

**DOI:** 10.3390/nu11102374

**Published:** 2019-10-05

**Authors:** Yohannes Adama Melaku, Amy C. Reynolds, Tiffany K. Gill, Sarah Appleton, Robert Adams

**Affiliations:** 1 Adelaide Institute for Sleep Health, College of Medicine and Public Health, Flinders University, Bedford Park 5042, SA, Australia; sarah.appleton@flinders.edu.au (S.A.); robert.adams@flinders.edu.au (R.A.); 2 The Appleton Institute, CQ University Australia, Adelaide 5034, SA, Australia; a.reynolds@cqu.edu.au; 3 School of Health, Medical and Applied Sciences, CQ University Australia Adelaide Campus, Adelaide 5034, SA, Australia; 4 Adelaide Medical School, University of Adelaide, Adelaide 5005, SA, Australia; tiffany.gill@adelaide.edu.au; 5 The Health Observatory, Discipline of Medicine, The Queen Elizabeth Hospital Campus, University of Adelaide, Woodville, SA 5011, Australia; 6 Freemason’s Centre for Men’s Health, Discipline of Medicine, The University of Adelaide, Adelaide 5005, SA, Australia

**Keywords:** fat, saturated fat, unsaturated fat, carbohydrate, protein, substitution analysis, sleepiness, excessive daytime sleepiness

## Abstract

Epidemiological evidence on the association between macronutrient intake and excessive daytime sleepiness (EDS) is scarce. Using data from the North West Adelaide Health Study, we aimed to determine the association between iso-caloric substitution of macronutrients and EDS. Data from 1997 adults aged ≥ 24 years were analyzed. Daytime sleepiness was measured using the Epworth Sleepiness Scale, a score ≥ 11 was considered EDS. Dietary intake data were collected using a food frequency questionnaire. We determined absolute and relative energy intake based on consumption of saturated and unsaturated fats, protein, and carbohydrate. Odds ratios (ORs) were used to determine the associations using log-binomial logistic regression with and without iso-caloric substitution methods, and models were adjusted for confounders. The prevalence of EDS in the sample was 10.6%. After adjusting for potential confounders, substituting 5% energy intake from protein with an equal amount of saturated fat (OR = 1.57; 95% CI: 1.00–2.45) and carbohydrate (OR = 1.23; 95% CI: 0.92–1.65) increased the odds of EDS. When carbohydrate was substituted with saturated fat (OR = 1.27; 95% CI: 0.93–1.59), the odds of EDS were increased. The odds of EDS were lower when saturated fat was substituted with unsaturated fat (OR = 0.74; 95% CI: 0.51–1.06), protein (OR = 0.63; 95% CI: 0.41–0.99) or carbohydrate (OR = 0.79; 95% CI: 0.57–1.08). While these results were consistent over different iso-caloric substitution methods, inconsistent results were found with standard regression. While substitution of fat and carbohydrate with protein was inversely associated with EDS, substitution of protein with fat and carbohydrate was positively associated with EDS. Randomized trials are needed to confirm if dietary interventions can be used to improve daytime alertness in those with EDS.

## 1. Introduction

Excessive daytime sleepiness (EDS) is defined as a state of pressure or perceived need to sleep in different situations or activities when wakefulness should be anticipated [1]. Excessive daytime sleepiness is often an indicator of underlying chronic health conditions and is particularly recognized as an indicator of sleep problems [2]. The prevalence of EDS in the general population of Australia is about 15% [3,4] and it is associated with health and societal consequences [5] including increased risk of work-related errors and injuries [5,6,7] and cardiovascular diseases and mortality [8]. Major factors associated with EDS are older age [9], short sleep duration [10], low physical activity [11], obesity [12], sleep apnea [3], depression [13], diabetes [14], and kidney disease [15]. Increasingly, a relationship between diet and EDS is recognized. In addition to contributing to regulation of the circadian rhythms [16] and affecting sleep quality [17], diet has been linked with daytime alertness [18].

In controlled laboratory experimental settings, a high intake of saturated fat and carbohydrate is associated with increased daytime sleepiness and fatigue [12,19] and postprandial sleepiness [20]. This is likely to be partially due to the less restorative sleep and more arousals associated with poor diet quality [21]. However, experimental studies on diet are usually used to determine the acute effects of food and nutrients on sleep. Therefore, the association of habitual intake of food and nutrients with health outcomes in such studies is unclear [22], as most experimental studies have small sample sizes and short follow-up time.

Epidemiological evidence on the association between macronutrient intake and EDS is scarce. Limited previous studies have shown that high intake of total fat and carbohydrate could be associated with increased risk of nocturnal sleep disturbance [17,23], high apnea–hypopnea index and daytime sleepiness [18]. While these associations have been reported, no existing epidemiological studies have used the iso-caloric substitution method [24]—an approach used to estimate the independent effect of nutrients by accounting for correlation among nutrients. In addition, the study on macronutrient intake and excessive daytime sleepiness did not use a standard tool to measure EDS and was limited to men only [18].

We hypothesized that utilization of iso-caloric substitution analysis using epidemiological data provides more plausible and accurate associations between macronutrients and EDS, which can be directly translated into public health and nutritional interventions. Therefore, we conducted a large-scale epidemiological investigation of the associations between iso-caloric substitution of macronutrients and EDS among community dwelling men and women that, to best of our knowledge, is the first of its kind.

## 2. Materials and Methods

### 2.1. Study Design and Population

**Figure 1:**
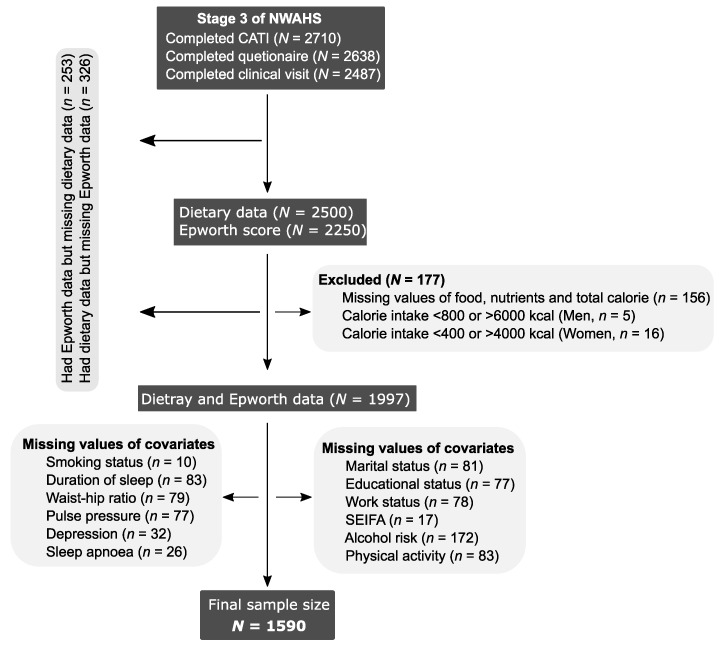
Sampling scheme. CATI—computer assisted telephone interview; NWAHS—North West Adelaide Health Study; SEIFA—socio-economic index for area.

Detailed methods about the North West Adelaide Health Study (NWAHS) can be found elsewhere [25,26]. In brief, we used longitudinal data from the NWAHS, a community-based study in the northern and western part of Adelaide. Up till now, the NWAHS has had four stages of data collection (Stage 1, 1999–2003; Stage 2, 2004–2006; Stage 3, 2008–2010; and North West 2015 (NW15), 2015). All randomly selected households with a telephone connected and a telephone number listed in the electronic White Pages (EWPs) were originally eligible for selection in the study. Within each household, the person who had their birthday last and was aged 18 years and over was selected for an interview and invited to attend the clinic for a biomedical examination. At Stage 1, a total of 4056 participants completed a self-administered questionnaire, computer-assisted telephone interview (CATI), and clinical visits. At Stage 3, data on diet (*n* = 2500) and daytime sleepiness score (*n* = 2250) were collected. In this study, we used data from the 1590 participants who had complete information on diet, Epworth Sleepiness Scale (ESS) score, and other covariates (Figure 1).

### 2.2. Assessment of Dietary and Nutrient Intakes

The Dietary Questionnaire for Epidemiological Studies (DQES-V3.1) developed by the Cancer Council of Victoria was used to assess self-reported intake of food and beverages over the previous year. The different versions of the questionnaire have been validated, and this tool is most commonly used in epidemiological studies [27,28]. Total daily food and nutrient intake was calculated using the Australian NUTTAB 95 (Food Standards Australia New Zealand, Canberra, 1995) food composition database. For each participant, the amount of macronutrients (i.e., fat, protein, and carbohydrate) consumed per day was calculated in grams.

### 2.3. Outcome Variable

The Epworth sleepiness scale (ESS) was used to assess daytime sleepiness [29]. The ESS has eight questions (components) that determine the chance of dozing in different situations (sitting and reading; watching television; sitting, inactive in a public place; as a passenger in a car for an hour without a break; lying down to rest in the afternoon when circumstances permit; sitting and talking to someone; sitting quietly after a lunch without alcohol; in a car, while stopped for a few minutes in traffic) with four responses (0 = would never doze; 1 = slight chance of dozing; 2 = moderate chance of dozing; and 3 = high chance of dozing). The score ranges from 0 to 24 and an ESS score of at least 11 was considered as EDS [30]. The ESS is a reliable measure of daytime sleepiness with high internal consistency [31] and is commonly used in large cohorts [32].

### 2.4. Covariates

At Stage 3, data on socio-demographic, lifestyle, physical measurements, and chronic conditions were collected. Marital status was classified as married/living with partner, separated/divorced, widowed, and never married. Highest educational achievement (did not complete school/high school level, trade/certificate/diploma, and degree and higher) and employment status (employed, unemployed, retired, and other) of participants were assessed. The Socio-Economic Index for Area (SEIFA) is an index determined by the Australian Bureau of Statistics that ranks areas in Australia according to relative socio-economic advantage and disadvantage. The values were divided into quintiles, with the highest representing greatest advantages [33].

Lifestyle factors included smoking, alcohol consumption, physical activity levels, and duration of sleep (hours). We categorized smoking status as non-smokers, ex-smokers, and current smokers. Alcohol risk was determined based on the 1989 National Heart Foundation Risk Factor Prevalence study classification [34] and was categorized as non-drinkers, low risk, and intermediate to very high risk. Physical activity was assessed using the Active Australia Survey questionnaire [35]. Waist and hip circumferences were measured in centimeters using flexible plastic tape to the nearest 0.1 cm. The tape was maintained on a horizontal plane, with the participant in a standing position. Waist circumference was taken at the midpoint between the lower margin of the last rib and the top of the iliac crest. Hip circumference was taken around the widest portion of the buttocks. Three measurements were taken, and the mean was calculated for each. The waist-to-hip ratio, the waist circumference divided by the hip circumference, was determined and used in the analysis as it was found to be more predictive of cardiovascular disease than waist circumference in previous study [36]. Pulse pressure was calculated as mean systolic pressure (mmHg) minus mean diastolic pressure (mmHg). Data on self-reported doctor diagnosed sleep apnea was collected. Diabetes was determined based on fasting plasma glucose (FPG) greater than 7.0 mmol/L or self-reported doctor diagnosed diabetes. Depression symptoms were scored using the Center for Epidemiological Studies-Depression (CES-D) and participants with a CES-D score ≥ 16 were considered to be depressed [37].

### 2.5. Statistical Analysis

We used proportion and mean (±SD) to summarize the data descriptively. We calculated total energy intake (EI) per day in kcal from fat (1 g = 9 kcal), carbohydrate (1 g = 4 kcal), and protein (1 g = 4 kcal). The relative percentage of energy intake from these macronutrients was calculated ((EI from macronutrient/total EI) × 100%). We segregated total fat intake into saturated and unsaturated in the analysis. Quartiles of the EI from each macronutrient were determined.

To assess the associations of absolute consumption (in grams) and relative EI (percent) from macronutrient with EDS, the odds ratios (ORs) were determined using log-binomial logistic regression models using two approaches. In the first approach, the association of quartiles of EI (kcal) from each macronutrient with EDS was determined along with trend tests considering quartiles as continuous variables. In this approach, we developed two models: model 1 was adjusted for total EI; model 2 was additionally adjusted for sex, age (continuous), marital status, education, work status, SEIFA, waist-to-hip ratio (continues), sleep duration (in hours), exercise level, alcohol consumption, pulse pressure (continuous), sleep apnea, diabetes status, and depression.

In the second approach, the association between the substitution of one macronutrient by another and EDS was assessed using three methods as previously described [24]: partition, standard regression, and nutrient density methods. These approaches have been commonly used in epidemiological studies [38,39,40]. In the partition method, we used an increase in 10 g of macronutrient intake (to estimate the effect of both caloric and non-caloric components) and a 100 kcal increase in EI (to estimate the effect of caloric intake only) from each of the macronutrients. We included either absolute intake (per 10 g increase) or EI (per 100 kcal increase) from each of the macronutrients separately and concurrently in our multivariable model. The difference in the β coefficients for a specific macronutrient intake and the others was used to estimate the odds ratio and 95% confidence interval. The odds ratio was then interpreted as the association of reducing a specific macronutrient intake (by 10 g or 100 kcal) while increasing an equal amount in the other ones. Total EI was not part of the model specification [41].

In the standard regression method, we used an increase in absolute macronutrient intake by 10 g. We included total EI as a covariate in this model. In the nutrient density model, the relative proportions of EI in percent ((EI from a specific macronutrient/total EI) × 100%) from macronutrients were included so that the sum of all the components remains 100% and total EI was part of the model [24]. In this study, the substitution model was performed considering a 5% increase in EI from a specific macronutrient. In both standard regression and nutrient density methods, one of the macronutrients (saturated fat, unsaturated fat, protein or carbohydrate) was removed from the model to estimate the substitution effect by other macronutrients specified in the model.

We developed two models: model 1 was adjusted for no covariates (partition method) and for total energy intake (standard regression and nutrient density methods); model 2 was additionally adjusted for sex, age (continuous), marital status, education, work status, SEIFA, waist-to-hip ratio (continues), sleep duration, alcohol intake, exercise level, pulse pressure (continuous), sleep apnea, diabetes status, and depression. We assessed the extent of (indirect) association between replacing protein with saturated fat and EDS (nutrient density method) mediated by depression and waist-to-hip ratio using a method proposed by Baron and Kenny [42].

Further, we assessed the association of substituting saturated fat and protein with each of the ESS components using ordinal logistic regression using nutrient density method (model 2). Statistical analyses were performed using Stata version 15.1 (Stata Corporation, College Station, TX, USA) and R version 3.1.0 (R Foundation for Statistical Computing, Vienna, Austria).

## 3. Results

### 3.1. Participants’ Characteristics

The mean age (SD) of the study participants was 56.3 (13.9) years. Almost half (48.1%) of the participants’ highest education level was high school or below, and a quarter (24.6%) were in the lowest quintile (most disadvantaged) of SEIFA. Half (49.4%) of the participants had low alcohol risk or were non-drinkers and just over one-third (37.1%) had sufficient physical activity. The mean (SD) duration of sleep was 7.2 (1.2) hours and 6% had self-reported sleep apnea. The mean (SD) total calorie intake (from fat, carbohydrate, and protein) was 1984.5 (591.4) and mean EI percentage from unsaturated fat, saturated fat, carbohydrate, and protein was 26.6%, 12.8%, 41.3%, and 19.3%, respectively. The prevalence of EDS was 10.6% (Table 1).

### 3.2. Association between EI and EDS

**Figure 2:**
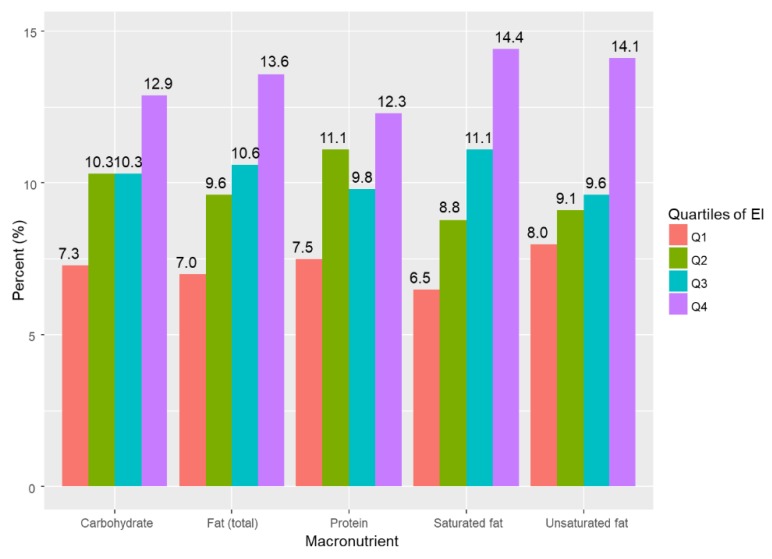
Prevalence of excessive daytime sleepiness by quartiles of energy intake from macronutrients (Q1, lowest energy intake; Q2, highest energy intake).

The prevalence of EDS across the quartiles of EI from specific macronutrient is depicted in Figure 2. The prevalence of EDS increased across the quartiles of EI from saturated fat (*p* = 0.002), total fat (*p* = 0.022), and unsaturated fat (*p* = 0.025).

There was a 77% (OR = 1.77; 95% CI: 0.87–3.64) increase in odds of EDS among participants in the fourth (highest) quartile of calories from saturated fat compared to those in the first quartile (model 2). The odds of EDS were 42% (OR = 1.42; 95% CI: 0.62–3.25) higher among participants in the fourth quartile of calories from carbohydrate compared to those in the first quartile. A higher intake of protein was not associated with EDS (Table 2).

### 3.3. Substitution Models

Table 3 shows EDS associated with an increase of 10 g (absolute intake), 100 kcal or 5% EI from a specific macronutrient per day and a simultaneous decrease (equal amount) of another macronutrient using three substitution models (partition, standard regression, and nutrient density models). In all three methods, results showed a substantially higher odds of EDS associated with an increase in saturated fat intake and a simultaneous decrease in protein intake (model 2). A 100 kcal increase in EI from saturated fat per day and a concurrent similar amount of EI decrease in protein increased the odds of EDS by 49% (OR = 1.49; 95% CI: 0.98–2.26) (partition model). A 10 g increase in saturated fat per day and a concurrent decrease of a similar amount in protein intake increased the odds of EDS by 43% (OR = 1.43; 95% CI: 0.98–2.08) (standard regression model). A 57% (OR = 1.57; 95% CI: 1.00–2.45) increase in the odds of EDS was found to be associated with a 5% increase of EI from saturated fat and a simultaneous decrease in EI from protein. Substituting 5% of EI from protein with carbohydrate increased odds of EDS by 23% (OR = 1.23; 95% CI: 0.92–1.65) (model 2, nutrient density model).

We found an increase in odds of EDS associated with replacing carbohydrate with saturated fat in all substitution models. The odds of EDS associated with the substitution of 5% of EI from carbohydrate by protein decreased by 19% (OR = 0.81; 95% CI: 0.61–1.09) (model 2, nutrient density model). In general, a decrease in saturated fat consumption and a concurrent increase of other macronutrients was associated with reduced odds of EDS across all substitution models. Substitution of 10 g of saturated fat with the same amount of protein resulted in a 25% (OR = 0.75; 95% CI: 0.56–1.00) reduction in odds of EDS (model 2, partition model). A 5% decrease in EI from saturated fat and a simultaneous increase of EI from protein was inversely associated with EDS (OR = 0.74; 95% CI: 0.51–1.06) (model 2, nutrient density model). Substitution of unsaturated fat by saturated fat increased the odds of EDS in all substitution models (Table 3).

The association between replacing 5% of energy from protein with saturated fat and EDS (total effect, OR = 1.68; 95% CI: 1.08–2.61) that was mediated by depression was 22% (OR = 1.15; 95% CI: 1.00–1.62) (model 2, nutrient density model). We did not find an association between saturated fat and EDS that was mediated by waist-to-hip ratio.

**Figure 3:**
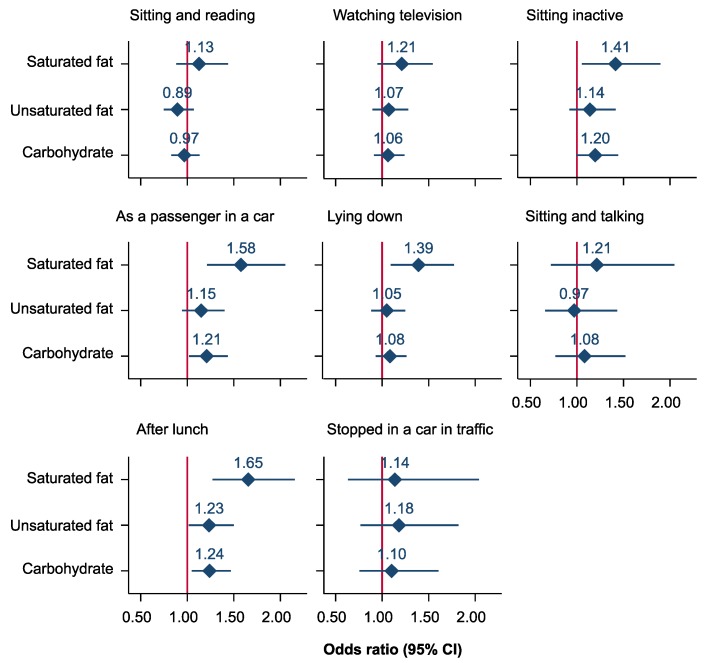
Odds ratios (95% confidence intervals) for excessive daytime sleepiness associated with a 5% decrease of energy per day from protein and a simultaneous 5% increase of energy per day from saturated fat, unsaturated fat, and carbohydrate in the North West Adelaide Health Study. (Nutrient density model. The model was adjusted for total energy intake from fat, carbohydrate, protein, sex, age (continuous), marital status, education, work status, Socio-Economic Indexes for Areas, waist-to-hip ratio (continues), alcohol intake, exercise level, sleep duration, pulse pressure (continuous), sleep apnea, diabetes status, and depression.).

**Figure 4:**
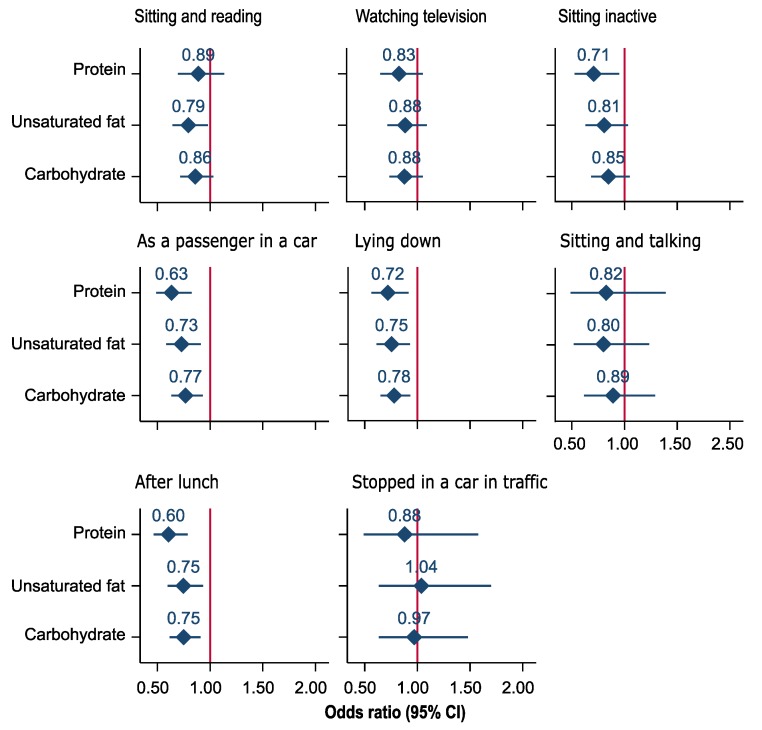
Odds ratio (95% confidence intervals) for excessive daytime sleepiness associated with a 5% decrease of energy per day from saturated fat and a simultaneous 5% increase of energy per day from protein, unsaturated fat, and carbohydrate in the North West Adelaide Health Study. (Nutrient density model. The model was adjusted for total energy intake from fat, carbohydrate, protein, sex, age (continuous), marital status, education, work status, Socio-Economic Indexes for Areas, waist-to-hip ratio (continues), alcohol intake, exercise level, sleep duration, pulse pressure (continuous), sleep apnea, diabetes status, and depression.).

Associations of iso-caloric substitution of saturated fat and protein with individual components of ESS using the nutrient density model are depicted in Figure 3 and Figure 4, respectively. We found that iso-caloric substitution of protein with saturated fat increased the odds of an individual reporting daytime sleepiness while sitting inactive and lying down, during travel as a passenger, and after lunch. Substitution of protein with carbohydrate increased the odds of daytime sleepiness while sitting inactive, during travel as a passenger, and after lunch (Figure 3). In general, the substitution of saturated fat by protein, unsaturated fat, and carbohydrate was inversely associated with daytime sleepiness for all components of ESS except stopped in a car in traffic for few minutes (Figure 4).

## 4. Discussion

To the best of our knowledge, this study is the first to assess the association of iso-caloric substitution of macronutrients with EDS using population-based data. Increasing EI from saturated fat was positively associated with EDS, with modest similar effect from carbohydrate. The EI from protein was inversely associated with EDS. In addition, we demonstrated that EI from macronutrients and most individual components of the ESS were associated consistently. The use of iso-caloric substitution analyses provided a more consistent estimate of associations between EI from macronutrients and EDS.

### 4.1. Comparison with Other Studies

Epidemiological studies of the association between fat intake and EDS are scarce and the findings are conflicting. Our previous work [18] showed that total fat intake was associated with self-reported daytime sleepiness among men aged 35–80 years. However, the study did not examine the association between saturated fat and daytime sleepiness and did not investigate the substitution effect of macronutrients [18]. This limits the application of findings to real-world dietary interventions, as it is not clear which food types to target for change. The current study found that saturated fat, but not unsaturated fat, was positively associated with EDS, and there was no association with total fat intake. In an experimental study, it has been shown that high saturated fat intake, but not unsaturated fat intake, was associated with poor quality sleep, particularly reduced slow wave sleep [21], which may contribute to EDS. Similarly, another study showed that total fat intake was not associated with insomnia symptoms [23]. In addition, a study by Wells et al. [20] demonstrated that increased daytime sleepiness was associated with high-fat diets. We showed that the association between unsaturated fat and EDS depended on the type of macronutrient to be substituted. While substituting protein with an equal amount of unsaturated fat increased the odds of EDS, replacing saturated fat with unsaturated fat decreased the odds of EDS. The current study aligns with findings from small-scale experimental studies [21,23], which suggest that saturated fat (not total fat) is an important component of diet that increases EDS. This is likely an important consideration for diet strategies to reduce daytime sleepiness.

Regarding carbohydrate intake, studies have reported conflicting findings on its association with sleep quality [18,23,43,44]. A cross-sectional study among Japanese females reported that high carbohydrate meals, including energy drinks and sugar-sweetened beverages, were associated with self-reported poor-quality sleep [43]. In another study, low carbohydrate intake was inversely associated with difficulties maintaining sleep [23]. On the other hand, carbohydrate was not associated with self-reported daytime sleepiness in a cohort of Australian men [18]. These conflicting findings could be due to the differences in carbohydrate quality. A study by St-Onge et al. [21] showed that low-fiber and high-sugar intake was associated with less restorative sleep and more arousal which could lead to EDS. In addition, experimental studies have shown that high glycemic index and carbohydrate diets were associated with an increased subjective sleepiness [20,45]. In our study, the data did not allow us to examine the difference in the effect of carbohydrate quality on EDS, which may explain the modest positive association between carbohydrate and EDS in study findings.

We found that EI from protein was inversely associated with EDS, in particular when substituting protein for saturated fat. These findings contrast with a cross-sectional study among men which showed an increased risk of daytime sleepiness associated with increased intake of protein [18]. The study did not use iso-caloric substitution analysis methods to determine the association between protein and daytime sleepiness. As a result, the findings [18] could be biased due to the high correlation among macronutrients. Conversely, Tanaka et al. [23] reported that low protein intake was associated with poor-quality nocturnal sleep. Further support for reduced sleepiness with higher protein intake is seen from a clinical study among 44 participants aged 19–22 years, which demonstrated a reduction in wake episodes after consumption of a high protein diet [46]. The current study, when considered with findings from experimental studies with short follow-ups, suggests high protein intake and low intake of saturated fat and carbohydrate may be important for patients with EDS to improve daytime alertness.

### 4.2. Potential Mechanisms and Implications

Two potential mechanisms, direct (acute) or indirect, may explain why EI from macronutrients could affect daytime sleepiness. The direct effect of EI from macronutrients on daytime sleepiness could be due to acute changes in hormone and neuroendocrine signaling [20,47]. In our study, we observed the strongest association between EI from macronutrients and an increased odds of sleepiness for the ESS question relating to the likelihood of falling asleep after a meal. This may suggest that specific macronutrient intake may be associated with alertness and concentration having an immediate effect following consumption. Similarly, previous experimental studies showed an association of various macronutrient intakes and difference in postprandial alertness and concentration. Substitution of fat with carbohydrate while keeping total energy constant at lunch time has also been shown to result in a reduction in alertness and concentration [48]. A study by Wells et al. [20] reported increased plasma insulin and cholecystokinin (CCK), a satiety hormone, after an intake of low-fat–high-carbohydrate and high-fat–low-carbohydrate meals, respectively. Increased insulin in the blood and CCK are associated with reduced alertness. A review by Panossian et al. [12] showed that the acute effect of high-fat and carbohydrate intake on the blood (increased glucose), pancreas (increased insulin), and the gut (increased enterostatin, increased leptin, decreased ghrelin, increased CCK, and increased peptide YY) could be responsible for immediate effects on EDS observed in clinical studies. These metabolites contribute to sedation by modulating wake neuron signaling, orexin, serotonin and norepinephrine [12]. Furthermore, increased plasma insulin as a result of high glycemic index meals was associated with upregulation of tryptophan (precursor of melatonin and serotonin) [49] and insulin which increase EDS in synergy [12,17].

The indirect effect of diet on daytime sleepiness could manifest in two ways: by routinely affecting nocturnal sleep quality [17] and by contributing to long-term metabolic consequences that in turn increase the propensity for EDS. Fat and carbohydrate intake have different effects on different stages and quality of sleep. When there is a high intake of fat, CCK is released by the duodenum [50]. It has previously been demonstrated that CCK hormone promotes slow wave sleep (stage 3 sleep) and non-rapid eye movement (NREM) sleep [51]. In humans, increased CCK and subjective fatigue were positively associated with a high-fat diet [19]. In addition, carbohydrate intake was positively associated with percentage of the stage 2 sleep, rapid eye movement (REM) sleep latency, sleep onset latency, and wake after sleep onset, and negatively associated with sleep efficiency (SE) and REM sleep [52]. A high intake of carbohydrate is associated with increased tryptophan which shorten sleep onset [45]. Although high carbohydrate consumption shortened sleep onset [46], subsequent sleep quality, such as slow wave sleep, and arousals during sleep could be severely compromised [53,54]. When experienced chronically, this may explain why participants with poorer diet report having EDS.

Since we collected habitual dietary intake, the association of macronutrient intake (particularly saturated fat and carbohydrate) and EDS in this specific study could also be a result of the long-term impact of high EI from fat and carbohydrate that eventually leads to an increased risk of adiposity and abnormal metabolic conditions which are in turn associated with EDS [55]. Adiposity is associated with EDS [12], and this association be by affecting nocturnal sleep quality [56,57] and/or as a result of upregulating inflammatory markers and hormones, such as tumor necrosis factor alpha (TNFa) [58], interleukin 6 (IL-6) [59], and leptin [60]. These markers are associated with decreased alertness during the daytime [12]. On the other hand, diet is the major contributor to the burden of chronic diseases [61]. In particular, high EI from saturated fat and carbohydrate is associated with an increased risk of cardiovascular diseases [62], diabetes [63], and mental health problems, such as depression [64]. These diet-related diseases are in turn associated with increased risk of EDS [14]. In the current study, 22% of the association between saturated fat and EDS was mediated by depression. However, this should be confirmed using longitudinal studies.

The current study, along with previous related investigations [12,17], demonstrated that dietary interventions, particularly the substitution of food types, could be used to support reductions in EDS and associated consequences. Impaired alertness is a prominent symptom in some population groups such as OSA patients, patients with insomnia, obese individuals, and shift workers, resulting in increased risk of injury, work-related errors, and compromised quality of life [5]. Dietary interventions could be a feasible and effective approach to alleviate EDS in many at-risk groups, and likely complimentary to existing alternative interventions including education, pharmacotherapy, and other lifestyle interventions. Standard treatments for underlying conditions related to EDS, such as continuous positive airway pressure for OSA patients, may not necessarily improve daytime sleepiness [65,66], in which case dietary interventions could be useful. Similarly, the substitution approach for dietary intake may be a valuable tool for shift-workers who are unable to alter the timing of work schedules and consequently, food intake. More work is warranted at a population level in this area, particularly around the timing of energy intake. In this endeavor, not only EI but also timing, frequency, and regularity of meals that may potentially affect EDS, either directly or indirectly, should be the focus of future studies. Our results form the basis for designing clinical trials of dietary interventions with substitution of food types with different levels of macronutrients.

Interpretation of the findings in this study should consider some methodological limitations. First, the food frequency questionnaire has drawbacks (such as recall bias) in measuring dietary intake, although the tool is widely used to measure usual intake of food and nutrients in large epidemiological studies [67]. Second, although daytime sleepiness was measured using the most commonly used self-reported standardized instrument (ESS) [29], measurement bias could be introduced affecting the association between EI and EDS towards null. Third, residual confounding could bias our findings. Fourth, a high number of cases with missing values was another limitation. Fifth, we did not have data on timing, frequency, and regularity of meal which is shown to have an impact on circadian rhythms [68] and which, ultimately, can impair daytime alertness. Sixth, causality cannot be established as we used cross-sectional data.

## 5. Conclusions

In summary, saturated fat intake was positively associated with EDS, with a modest similar association of carbohydrate intake. However, protein was inversely associated with EDS. This study highlights the important role of diet, particularly relative EI from macronutrients, in predicting EDS among adults. Clinical and public health interventions aimed at lowering EDS and associated consequences should consider dietary approaches as important strategies at the individual and population levels. People who have sleep-related disorders (such as OSA patients) and disturbed circadian rhythm (such as shift-workers) may benefit from dietary interventions to alleviate EDS. Our findings can assist in the design of trials involving substitution of food types in populations with EDS. We found that substitution analysis is an important and plausible tool to understand the role of macronutrients and EI in EDS. Longitudinal epidemiological studies are needed to examine the contribution of macronutrients and associated EI to EDS. In addition, the role of timing, regularity, and frequency of diet and EI from macronutrients remains to be investigated in population studies.

## Figures and Tables

**Table 1:** Characteristics of study participants (*N* = 1997).

Variable	Category	*N* (%)
Sex	Male	930 (46.6%)
Age (years) ^#^		56.3 (13.9)
Marital status	Married or living with partner	1332 (66.7%)
	Separated/divorced	256 (12.8%)
	Widowed	164 (8.2%)
	Never married	164 (8.2%)
	Missing	81 (4.1%)
Educational status	Did not complete school/high school level	961 (48.1%)
	Trade/certificate/diploma	614 (30.7%)
	Degree or higher	345 (17.3%)
	Missing	77 (3.9%)
Work status	Employed	1103 (55.2%)
	Unemployed	29 (1.5%)
	Retired	605 (30.3%)
	Other	182 (9.1%)
	Missing	78 (3.9%)
SEIFA	Lowest quintile	498 (24.9%)
	Low quintile	491 (24.6%)
	Middle quintile	432 (21.6%)
	High quintile	426 (21.3%)
	Highest quintile	133 (6.7%)
	Missing	17 (0.9%)
Alcohol risk	Nondrinkers, no risk	987 (49.4%)
	Low risk	744 (37.3%)
	Intermediate to very high risk	94 (4.7%)
	Missing	172 (8.6%)
Physical activity level	No activity	355 (17.8%)
	Activity but not sufficient	819 (41.0%)
	Sufficient activity	740 (37.1%)
	Missing	83 (4.2%)
Smoking status	Non-smoker	926 (46.4%)
	Ex-smoker	774 (38.8%)
	Current smoker	287 (14.4%)
	Missing	10 (0.5%)
Sleeping duration (hours per day) ^#^		7.2 (1.2)
Waist-to-hip ratio ^#^		0.89 (0.09)
Pulse pressure (mmHg) ^#^		50.6 (14.3)
Diabetes	Yes	191 (9.6%)
Depression	Yes	338 (16.9%)
	Missing	32 (1.6%)
Sleep apnea	Yes	119 (6.0%)
	Missing	26 (1.3%)
Total energy in kcal ^#^ (fat, carbohydrate, and protein)		1984.5 (591.4)
Energy from unsaturated fat ^#^ (%)		26.6 (5.4)
Energy from saturated fat ^#^ (%)		12.8 (2.7)
Energy from carbohydrate ^#^ (%)		41.3 (7.0)
Energy from protein ^#^ (%)		19.3 (3.3)
Excessive daytime sleepiness		211 (10.6%)

^#^ Data are presented as mean (SD). SEIFA—Socio-Economic Indexes for Areas; PAL—physical activity level.

**Table 2:** Odds ratios (95% confidence intervals) for excessive daytime sleepiness associated with quartile of energy from macronutrients (per day) in the North West Adelaide Health Study.

Model	Odds Ratio (95% CI)				
	Q1	Q2	Q3	Q4	*p* Trend
**Total fat**					
Model 1	1.00	1.32 (0.78–2.25)	1.39 (0.78–2.49)	1.72 (0.86–3.45)	0.147
Model 2	1.00	1.21 (0.69–2.12)	1.11 (0.60–2.05)	1.06 (0.49–2.28)	0.975
**Saturated fat**					
Model 1	1.00	1.37 (0.80–2.36)	1.75 (0.99–3.10)	2.33 (1.20–4.52)	0.011
Model 2	1.00	1.26 (0.71–2.23)	1.52 (0.82–2.81)	1.77 (0.87–3.64)	0.106
**Unsaturated fat**					
Model 1	1.00	1.07 (0.64–1.78)	1.04 (0.60–1.81)	1.47 (0.80–2.73)	0.247
Model 2	1.00	0.92 (0.53–1.58)	0.86 (0.48–1.54)	0.97 (0.49–1.92)	0.899
**Carbohydrate**					
Model 1	1.00	1.30 (0.77–2.20)	1.17 (0.65–2.11)	1.24 (0.57–2.66)	0.695
Model 2	1.00	1.33 (0.76–2.32)	1.23 (0.65–2.32)	1.42 (0.62–3.25)	0.507
**Protein**					
Model 1	1.00	1.32 (0.80–2.19)	1.01 (0.57–1.78)	1.06 (0.54–2.09)	0.870
Model 2	1.00	1.47 (0.86–2.53)	1.04 (0.56–1.91)	1.03 (0.48–2.19)	0.761

Model 1 was adjusted for total EI; model 2 was additionally adjusted for sex, age (continuous), marital status, education, work status, the Socio-Economic Indexes for Areas, waist-to-hip ratio (continuous), alcohol intake, exercise level, sleep duration, pulse pressure (continuous), sleep apnea, diabetes status, and depression.

**Table 3:** Odds ratio (95% confidence intervals) for excessive daytime sleepiness associated with decrease of 10 g, 100 kcal or 5% per day of one of the macronutrients and simultaneous increase of 10 g, 100 kcal or 5% per day of other macronutrients in the North West Adelaide Health Study.

Model	Odds Ratio (95% CI)			
	Saturated Fat	Unsaturated Fat	Protein	Carbohydrate
**Substituting protein**				
**Partition model**				
**Absolute intake (10 g)**				
Model 1	1.40 (1.08–1.81)	1.11 (0.94–1.31)		1.07 (0.96–1.19)
Model 2	1.34 (1.00–1.78)	1.06 (0.88–1.27)		1.07 (0.96–1.19)
**Energy intake (100 kcal)**				
Model 1	1.57 (1.07–2.30)	1.22 (0.91–1.63)		1.17 (0.92–1.50)
Model 2	1.49 (0.98–2.26)	1.14 (0.83–1.58)		1.18 (0.90–1.53)
**Standard regression model (10 g)**				
Model 1	1.50 (1.07–2.12)	1.20 (0.92–1.55)		1.07 (0.97–1.18)
Model 2	1.43 (0.98–2.08)	1.13 (0.85–1.50)		1.07 (0.96–1.18)
**Nutrient density model (5% energy)**				
Model 1	1.74 (1.16– 2.63)	1.26 (0.92– 1.71)		1.24 (0.94–1.62)
Model 2	1.57 (1.00–2.45)	1.16 (0.83–1.62)		1.23 (0.92–1.65)
**Substituting carbohydrate**				
**Partition model**				
**Absolute intake (10 g)**				
Model 1	1.31 (1.06–1.63)	1.04 (0.93–1.17)	0.94 (0.85–1.03)	
Model 2	1.25 (0.98–1.60)	0.99 (0.87–1.13)	0.94 (0.84–1.04)	
**Energy intake (100 kcal)**				
Model 1	1.34 (1.04–1.72)	1.04 (0.90–1.19)	0.85 (0.67–1.09)	
Model 2	1.27 (0.96–1.67)	0.97 (0.84–1.13)	0.85 (0.65–1.11)	
**Standard regression model (10 g)**				
Model 1	1.30 (1.04–1.63)	1.03(0.92–1.17)	0.94(0.85–1.03)	
Model 2	1.24 (0.96–1.59)	0.98(0.85–1.12)	0.94(0.84–1.04)	
**Nutrient density model (5% energy)**				
Model 1	1.41 (1.06–1.88)	1.01 (0.87–1.19)	0.80 (0.62–1.06)	
Model 2	1.27 (0.93–1.75)	0.94 (0.79–1.12)	0.81 (0.61–1.09)	
**Substituting saturated fat**				
**Partition model**				
**Absolute intake (10 g)**				
Model 1		0.80 (0.61–1.04)	0.71 (0.55–0.93)	0.76 (0.61–0.95)
Model 2		0.79 (0.58–1.07)	0.75 (0.56–1.00)	0.80 (0.63–1.02)
**Energy intake (100 kcal)**				
Model 1		0.78 (0.57–1.05)	0.64 (0.43–0.93)	0.75 (0.58–0.96)
Model 2		0.77 (0.55–1.08)	0.67 (0.44–1.02)	0.79 (0.60–1.05)
**Standard regression model (10 g)**				
Model 1		0.80 (0.61–1.04)	0.83 (0.72–0.97)	0.89 (0.80–0.98)
Model 2		0.79 (0.58–1.07)	0.85 (0.72–1.01)	0.91 (0.81–1.02)
**Nutrient density model (5% energy)**				
Model 1		0.72 (0.52–1.01)	0.57 (0.38–0.86)	0.71 (0.53–0.94)
Model 2		0.74 (0.51–1.06)	0.63 (0.41–0.99)	0.79 (0.57–1.08)
**Substituting unsaturated fat**				
**Partition model**				
**Absolute intake (10 g)**				
Model 1	1.26 (0.96–1.65)		0.90 (0.76–1.06)	0.96 (0.86–1.07)
Model 2	1.27 (0.93–1.71)		0.95 (0.79–1.14)	1.01 (0.89–1.15)
**Energy intake (100 kcal)**				
Model 1	1.29 (0.95–1.74)		0.82 (0.61–1.10)	0.96 (0.84–1.10)
Model 2	1.30 (0.93–1.82)		0.87 (0.64–1.20)	1.03 (0.88–1.20)
**Standard regression model (10 g)**				
Model 1	1.26 (0.96–1.65)		0.92 (0.82–1.04)	0.98 (0.93–1.04)
Model 2	1.27 (0.93–1.71)		0.95 (0.83–1.08)	1.01 (0.95–1.07)
**Nutrient density model (5% energy)**				
Model 1	1.38 (0.99–1.93)		0.79 (0.58–1.08)	0.98 (0.84–1.15)
Model 2	1.36 (0.94–1.95)		0.86 (0.62–1.21)	1.06 (0.90–1.26)

Model 1 was adjusted for total energy intake (standard regression and nutrient density methods), energy intake (100 kcal or 5%) from, or absolute intake (10 g) of saturated and unsaturated fat, carbohydrate, and/or protein; model 2 was additionally adjusted for sex, age (continuous), marital status, education, work status, Socio-Economic Indexes for Areas, waist-to-hip ratio (continues), alcohol intake, exercise level, sleep duration, pulse pressure (continuous), sleep apnea, diabetes status, and depression. The models represent an increase in the macronutrients on the top raw with a decrease in the substituting macronutrients.
